# Relationship between Serum Levels of OPG and TGF-****β**** with Decreasing Rate of BMD in Native Chinese Women

**DOI:** 10.1155/2013/727164

**Published:** 2013-02-06

**Authors:** Xi-Yu Wu, Yi-Qun Peng, Hong Zhang, Hui Xie, Zhi-Feng Sheng, Xiang-Hang Luo, Ru-Chun Dai, Hou-De Zhou, Xian-Ping Wu, Er-Yuan Liao

**Affiliations:** Institute of Metabolism and Endocrinology, The Second Xiangya Hospital of Central South University, 139 Renmin-Zhong Road, Changsha, Hunan 410011, China

## Abstract

The objective of this study was to investigate the relationship between serum levels of OPG, TGF-**β**1, and TGF-**β**2 and BMD decrease rate (BDR) in native Chinese women. This cross-sectional study was performed on 465 healthy native Chinese women aged 35–80 years. Serum levels of OPG, TGF-**β**1, and TGF-**β**2 were determined. BDR was measured by DXA at the posteroanterior spine, hip, and distal forearm. At all skeletal sites tested, there was a negative correlation between BDR and serum levels of both OPG (*r* = −0.122 to –0.230, all *P* = 0.007–0.000) and TGF-**β**2 (*r* = −0.100 to –0.173, all *P* = 0.029–0.000) and a positive correlation between BDR and serum TGF-**β**1 (*r* = 0.245 − 0.365, all *P* = 0.000). After adjustment for age and BMI, there were no statistically significant correlations between serum levels of OPG or TGF-**β**2 and BDR. However, statistically significant correlations between serum TGF-**β**1 and BDR at the lumbar spine and ultradistal forearm remained. Multiple linear regression stepwise analysis showed that serum OPG could explain 1.4–3.7% of BDR variation. Serum TGF-**β**1 was a positive determinant of BDR and could explain 5.3–13.3% of BDR variation.

## 1. Introduction

Osteoprotegerin (OPG), transforming growth factor-*β*1 (TGF-*β*1) and TGF-*β*2 are cytokines closely associated with bone metabolism [1, 2]. OPG is one of the key cytokines secreted by osteoblasts and plays an important role in the bone remodeling balance [3]. OPG knockout can result in severe osteoporosis in mice [4], and overexpression of OPG can lead to osteopetrosis in OPG-transgenic mice [2, 5]. In postmenopausal women, the circulating OPG level affects bone turnover, changes in bone mass, and the prevalence of vertebral fractures [6, 7]. TGF-*β* is very rich in bone tissue [8, 9]. The ratio of TGF-*β*1 and TGF-*β*2 is about 4 : 1, and there is 70% sequence homology between the two forms [10]. TGF-*β* could not only promote osteoblasts proliferation and differentiation [11, 12], but also inhibit osteoclasts activity [13, 14]. TGF-*β*1 gene knockout cause osteopenia in mice [15], while the mice with osteoporosis treated by TGF-*β* could increase BMD [16, 17]. TGF-*β*1 gene mutation can lead to low bone mass in human [18]. The studies demonstrated that both TGF-*β*1 and TGF-*β*2 stimulate bone formation and bone mineralization [19–21], but TGF-*β*2 was more active than TGF-*β*1 in stimulating formation of a mass [19]. *In vivo*, TGF-*β*2 stimulated synthesis of TGF-*β*1 in chondrocytes and osteoblasts [19]. TGF-beta may act as a bone-coupling factor linking bone resorption to bone formation [10]. TGF-*β* is expressed by osteoblasts; it not only stimulates the differentiation of osteoclasts and maintains their survival [22], but also regulates bone formation and resorption [23]. After menopause, serum TGF-*β*2 is increased in women with osteoporosis and shows a positive correlation with bone resorption markers [24].

Bone mineral density (BMD) decrease rate (BDR) [25] is an important parameter measured by dual-energy X-ray absorptiometry (DXA). When we used the Hologic DXA bone densitometer to measure BMD; BDR can be calculated by a peak reference (PR%) value in measurement report. Using the GE-Lunar DXA bone densitometer, the young adult mean (YAM%) represents the BMD. BDR means that compared to the peak BMD in reference databases, the percentage of BMD reducing or bone loss percentage in subjects. BDR is an important index closely related to the diagnosis of osteoporosis in Chinese [26] and Japanese [27]. The relationship between the cytokines OPG, TGF-*β*1 and TGF-*β*2 and BDR in native Chinese women remains unknown. To investigate this relationship, DXA was used to measure both BMD and BDR at the lumbar spine, proximal femur, and distal forearm in 465 healthy native Chinese women aged 35–80 years. Serum levels of OPG, TGF-*β*1, and TGF-*β*2 were also determined, and their relationships with BDR were analyzed.

## 2. Material and Methods

### 2.1. Study Participants

Four hundred and sixty-five healthy Chinese women aged 35–80 years were randomly selected between September 2007 and May 2010. These volunteers, all residents of Changsha and surrounding regions, were recruited by public health organizations (i.e., health stations/clinics) that provide health care for local residents. All subjects were screened by a detailed questionnaire, history, and physical examination. Women were excluded from the study if they had a condition affecting bone metabolism such as disease of the kidney, liver, parathyroid or thyroid, diabetes mellitus, oligomenorrhea or menopause at <40 years, hyperprolactinemia, oophorectomy, rheumatoid arthritis, ankylosing spondylitis, a malabsorption syndrome, a malignant tumor, hematologic disease or previous pathological fracture. Subjects were also excluded if they had been receiving glucocorticoids, estrogens, thyroid hormone, fluoride, bisphosphonate, calcitonin, thiazide diuretics, barbiturates, antiseizure medications, vitamin D- or calcium-containing drugs, as were those who smoked or consumed alcohol or caffeine. The study involved 142 premenopausal women, 58 perimenopausal women (last menses <12 months before the study started) and 265 postmenopausal women (last menses >12 months before the study started); of the latter, the mean (± SD) age at menopause was 48.3 ± 3.83 years (range 41–57 years) and the median duration of menopause was 11.0 years (range 1–40 years). The study was approved of the Ethical Committee of Xiangya Medical College, Central South University, China, and all participants provided written consent to participate.

### 2.2. Markers Measurement

Fasting morning (7–9 AM) blood samples were collected and centrifuged within 1 h and stored at −70°C until analysis. We measured serums TGF-*β*1 and TGF-*β*2 concentrations with a sensitive enzyme-linked immunosorbent assay (ELISA) kit (DRG International, Inc., Highway, Mountainside, NJ, USA) and OPG with an ELISA kit (Biomedica Gruppe, Vienna, Austria) and quantified the results using a *μ*Quant Universal Microplate Spectrophotometer (Bio-Tek Instruments, Inc., Winooski, VT, USA). The minimum detectable concentration was 0.14 pmol/L for OPG, 0.002 *μ*g/L for TGF-*β*1 and 0.01 *μ*g/L for TGF-*β*2. The intra- and interassay CVs were 6.4% and 8.2% for OPG, 3.8% and 8.8% for TGF-*β*1, and 5.9% and 8.9% for TGF-*β*2. BMD and BDR were measured with a DXA fan-beam bone densitometer (Hologic Delphi A; Hologic, Bedford, MA, USA) at the posteroanterior (PA) spine (L1–L4); the left hip, including the femoral neck (FN) and total hip; and the radius + ulna ultradistal (RUUD) of the nondominant forearm. The *in vivo* deviations in precision of two repeated BMD measurements in 33 subjects, determined by the root mean square coefficient of variation method [28], were 0.83% for the PA spine, 1.88% for the FN, 0.88% for the total hip, and 2.21% for the RUUD. A control spine phantom scan performed each day had a long-term (>13 years) coefficient of variation (CV) of <0.45%.

### 2.3. Statistical Analysis

All calculations were performed using SPSS V17.0 for Windows software (SPSS, Inc., Chicago, IL, USA). The geometric mean and SD were used for serums OPG, TGF-*β*1, and TGF-*β*2 because these did not follow a logarithmic normal distribution. All subjects were stratified by 10-year age groups, and serums OPG, TGF-*β*1, and TGF-*β*2, and BDR at the various skeletal sites were reported as the mean ± SD for each group. The mean values of the different parameters from each group were compared for significant differences and assessed using one-way analysis of variance whenever significant. The relationship between serums OPG, TGF-*β*1, and TGF-*β*2 with BDR at the various skeletal sites was evaluated by linear regression and Pearson's correlation analysis. BDR was calculated using the formula BDR(%) = subjects' PR(%) − 100% [25]. Peak BMD was calculated from the BMD reference database previously established and continuously improved by us [29]. Multiple linear regression was used to determine the influence of OPG, TGF-*β*1, and TGF-*β*2 on BDR.

## 3. Results

### 3.1. Basic Characteristics of the Subjects

The characteristics of the subjects are summarized in Table 1. Height and TGF-*β*1 were significantly higher in the premenopausal period than in the perimenopausal period and after menopause, whereas serum levels of OPG and TGF-*β*2 were markedly lower in the premenopausal period than in the perimenopausal period and after menopause. Height and weight were higher in the perimenopausal period than after menopause.  Table 2 shows the serum levels of the age-related cytokines and the BDR at the various skeletal sites. From 35 to 44 years, serums OPG and TGF-*β*2 were at their lowest levels, whereas TGF-*β*1 was at its highest. From 45 to 54 years, serum OPG was at its highest level. Above 45 years, there were no significant differences in TGF-*β*1 or TGF-*β*2 between any age group. Compared with the 35–44-year group, there was a significant increasing trend of BDR with age in subjects above 45 years of age. In subjects aged ≥65 years, the mean BDR at the RUUD was −34.1%, which was markedly higher than that at the other skeletal sites.

### 3.2. Correlations between BDR and Cytokines

**Figure 1:**
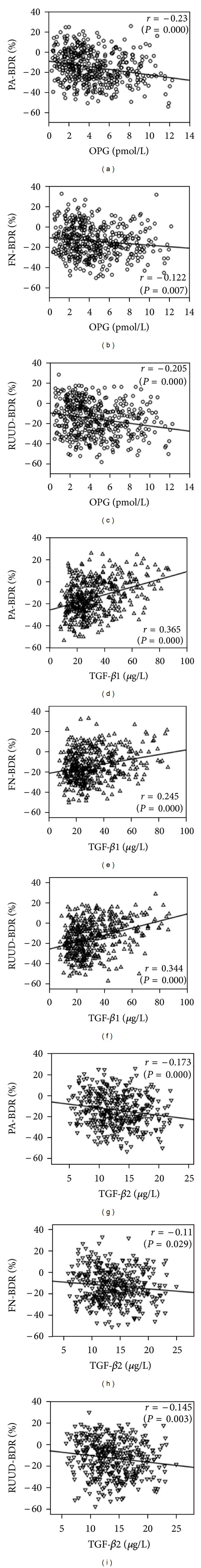
Scatter plots of serums OPG, TGF-*β*1, and TGF-*β*2 concentration versus BDR at various skeletal sites in native Chinese women. OPG: osteoprotegerin, TGF-*β*: transforming growth factor-beta, BDR: bone mineral density decrease rate, PA: posteroanterior spine, FN: femoral neck, and RUUD: radius + ulna ultradistal.

Figure 1 shows scatter plots and correlations between the cytokine levels and the BDR at the different skeletal sites. There were obvious negative correlations between serum levels of both OPG and TGF-*β*2 and BDR, and marked positive correlations between serum TGF-*β*1 and BDR.  Table 3 shows Pearson's correlation coefficients and partial correlation coefficients for the cytokines and BDRs at the different skeletal sites. There were notable positive correlations between serums OPG and TGF-*β*2, and marked negative correlations between serum TGF-*β*1 and serum levels of both OPG and TGF-*β*2. After controlling for age and body mass index (BMI), the partial correlation coefficients for both OPG and TGF-*β*2 with BDR were no longer statistically significant. However, the partial correlation coefficients for serum TGF-*β*1 with BDR at the PA spine and RUUD remained statistically significant.

### 3.3. Association between BDR and Cytokines

**Figure 2:**
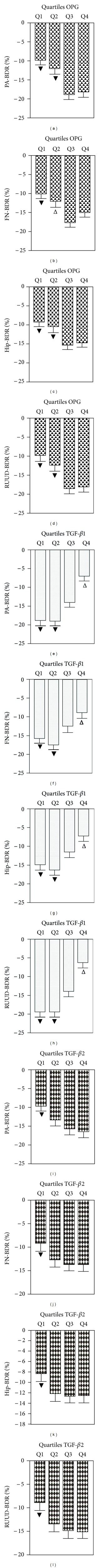
BDRs at different skeletal sites in native Chinese women displayed by quartiles of serums OPG, TGF-*β*1, and TGF-*β*2 concentrations. OPG: osteoprotegerin, TGF-*β*: transforming growth factor-beta, Q1: first quartile, Q2: second quartile, Q3: third quartile, Q4: fourth quartile, PA: posteroanterior spine, BDR: bone mineral density decreased rate, FN: femoral neck, Hip: total hip, and RUUD: radius + ulna ultradistal. ^▼^ *P* = 0.045–0.000 compared with Q3 and Q4; ^Δ^ *P* = 0.010–0.000 compared with Q3.

Figure 2 display comparisons between the cytokines. When serum OPG was grouped by quartile, the BDRs at the PA spine, hip, and RUUD in Q1 and Q2 were significantly higher than those in Q3 and Q4. At the FN, the mean BDR was lowest in Q3 and markedly lower than in Q1 and Q2. When serum TGF-*β*1 was grouped by quartile, the BDR in Q4 was notably higher than that in Q1, Q2, and Q3. The BDRs in Q1 and Q2 were lower, but there were no significant differences between them. When serum TGF-*β*2 was grouped according to quartile, the BDR was maximal in Q1, markedly higher than in Q2, Q3, and Q4.

Using serum levels of OPG, TGF-*β*1, and TGF-*β*2 as independent variables and the BDRs at the different skeletal sites as dependent variables, multiple linear regression stepwise analysis was conducted (Table 4). The results show that OPG could explain 1.4–3.7% of the variation in BDR at each skeletal sites. The influence of OPG on BDR was lowest at the FN (1.4%) and greatest at the RUUD (3.7%). TGF-*β*1 was a positive determinant of BDR at each skeletal site, explaining about 5.3–13.3% of BDR variation. The influence of TGF-*β*1 was lowest at the FN (5.3%) and greatest at the PA spine (13.3%). TGF-*β*2 had no influence on BDR at any skeletal site according to this analysis.

## 4. Discussion

Our research confirmed the presence of marked negative correlations between serum levels of both OPG and TGF-*β*2 and BDR in native Chinese women; thus, the BDR was lower with higher circulating levels of OPG or TGF-*β*2 and higher with lower levels of these cytokines. There was a notably positive correlation between serum TGF-*β*1 and BDR, indicating that the BDR was higher with higher circulating levels of TGF-*β*1 and lower with lower levels of this cytokine. The partial correlation coefficients for OPG and TGF-*β*2 levels with BDR were insignificant at all skeletal sites, suggesting that these correlations are affected by both age and BMI and weaken or disappear when these influences are excluded. The partial correlation coefficients for TGF-*β*1 and BDR at the PA spine and RUUD remained statistically significant, demonstrating that, though the correlations between TGF-*β*1 and BDR at these skeletal sites were affected by both age and BMI, they remained close. These findings also imply that the correlation between circulating TGF-*β*1 and BDR differed between the various skeletal sites.

The results illustrate that the serum levels of OPG were the highest in women aged 45–54 years because they are in the rapid bone loss period of early postmenopause (the average age of menopause is 48.3 ± 3.83 years in this group) (Table 2). The increasing serum levels of OPG may be a compensatory defense mechanism for resistance to rapid bone loss [30]. Previous research on the general population has shown that, after menopause, increased serum OPG is related to increased risks for osteoporosis and vertebral fracture in women [6]. However, Ueland et al. [31] found no correlations between OPG genetic polymorphisms or changes in serum OPG and morbidity from osteoporosis in elderly Australian women. Another study showed that serum OPG in women was positively correlated with bone turnover markers including TRACP-5b, osteocalcin, and C-terminal cross-linked telopeptide [32]. The authors suggest that circulating OPG might help to prevent bone mass loss in women. After menopause, the bone mass loss rate might be lower in women with higher OPG levels, thereby increasing the strength of the hip [33]. A longitudinal study with a large sample size found that bone loss rate in women was related to circulating OPG levels; similar to the results of our study, the higher the serum OPG, the greater the bone loss rate [34]. The main reason for inconsistent results demonstrated from different research groups may be related to the difference of race, age, and the sample quantity of subjects. Other studies have demonstrated that, in patients with hyperthyroidism [35] or enteritis [36], increased serum OPG might prevent excessive bone mass loss. Considering the finding that serum OPG is significantly decreased in adipose women in the perimenopausal period [37], the authors suggested that circulating OPG might have no protective effect on bone mass loss in these patients. Research on large samples of the general female population verified that common TGF-*β*1 genetic polymorphisms had no influence on various bone turnover markers, BMD, or bone mass loss [38]. Nonetheless, many studies have shown that changes of both TGF-*β*1 [18, 39–44] and TGF-*β*2 [24] are related to bone turnover velocity. For instance, serum TGF-*β*1 was increased and bone loss rate decreased in women with the TT genotype of the TGF-*β*1 gene [39], while the prevalence of brittle fracture was increased in women with the TC genotype [40]. Early studies demonstrated that TGF-*β*1 is a downstream factor of estrogen [13] and that TGF-*β*1 is involved in the vitamin D signaling pathway [45], which plays an important role in the local regulation of bone metabolism. New research suggests that TGF-*β*1 genetic polymorphism is associated with vitamin D and has an important effect on the incidence of osteoporotic vertebral fracture after menopause [46].

Our research demonstrated obvious differences in BDR related to cytokine levels. For serum OPG (Figure 2), BDRs at the PA spine, hip, and RUUD were significantly higher in Q1 and Q2 than in Q3 and Q4. For serum TGF-*β*1, the pattern of BDR was opposite to that for OPG; namely, BDR was minimal in Q1 and Q2 (lower TGF-*β*1) and maximal in Q4 (higher TGF-*β*1) at every skeletal site. The BDR decrease with TGF-*β*1 level increase is similar to other research results [15]. In TGF-*β*1 knockout mice, the bone mass in tibia metaphysis decreased by 30%, and BMD and bone strength decreased markedly [15]. Furthermore, the regulation of TGF-*β*1 levels in bone cells exhibited biphasic characteristics. Low concentrations of TGF-*β*1 and TGF-*β*2 increased the RANKL/OPG ratio due to the upregulation of these proteins in the osteoblasts/stromal cells and increased the differentiation of osteoclasts [47]. However, opposite effects were observed in the presence of high concentrations of TGF-*β*1 and TGF-*β*2 [47]. Recent studies have demonstrated that TGF-*β*1 is mainly expressed by differentiated osteoblasts and that it is deposited in the bone matrix [48]. TGF-*β*2, in contrast, is mainly expressed by the precursors of osteoblasts. These findings suggest that BDR in native Chinese women might be affected by changes in the circulating levels of cytokines including OPG, TGF-*β*1, and TGF-*β*2 and variations between different parts of the skeleton. We still found that TGF-*β*1 was independent determinant of BDR in our study population. TGF-*β*1 level could explain of 5.3–13.3% of the BDR variation. The influence of TGF-*β*1 on BDR was 2.9–4.6 times than that of OPG, with the two having opposite effects.

## 5. Conclusions

This study investigated correlations between serum levels of OPG, TGF-*β*1, and TGF-*β*2 and BDR at various skeletal sites in native Chinese women, with results indicating that changes in circulating TGF-*β*1 and OPG are related to BDR. TGF-*β*1 was a positive determinant of BDR.

## Figures and Tables

**Table 1:** Demographics and clinical characteristics of the study subjects.

Factor measured	Premenopausal (*n* = 142)	Perimenopausal (*n* = 58)	Postmenopausal (*n* = 265)
Age (years)	40.8 ± 3.50	48.0 ± 2.95	59.7 ± 7.30
Height (cm)	156.2 ± 5.38^b^	154.2 ± 5.05^c^	151.9 ± 4.54
Weight (kg)	56.9 ± 7.38^c^	57.1 ± 8.03^c^	54.3 ± 8.46
BMI (kg/m^2^)	23.3 ± 2.68	24.0 ± 3.03	23.5 ± 3.34
OPG (pmol/L)^a^	2.67 ± 1.86^b^	4.11 ± 1.96	4.40 ± 1.85
TGF-*β*1 (*μ*g/L)^a^	39.0 ± 1.60^b^	27.1 ± 1.67	24.0 ± 1.52
TGF-*β*2 (*μ*g/L)^a^	11.5 ± 1.28^b^	13.0 ± 1.36	14.3 ± 1.32

BMI: body mass index, OPG: osteoprotegerin, and TGF-*β*: transforming growth factor-beta.

^a^Values are geometric mean ± SD.

^b^ *P* = 0.045–0.000 compared with perimenopausal and postmenopausal.

^c^ *P* = 0.002–0.000 compared with postmenopausal.

**Table 2:** Age-related serums OPG, TGF-*β*1, and TGF-*β*2 and BDR at different skeletal sites in native Chinese women.

Parameter	Age (year)			
	35–44 (*n* = 128)	45–54 (*n* = 146)	55–64 (*n* = 117)	≥65 (*n* = 74)
OPG (pmol/L)^a^	2.61 ± 1.88^b^	4.76 ± 1.84^c^	4.09 ± 1.87	3.77 ± 1.88
TGF-*β*1 (*μ*g/L)^a^	38.9 ± 1.64^b^	25.2 ± 1.63	25.0 ± 1.52	24.9 ± 1.44
TGF-*β*2 (*μ*g/L)^a^	11.9 ± 1.32^b^	14.2 ± 1.33	14.0 ± 1.34	13.6 ± 1.31
PA-BDR (%)	−1.72 ± 11.3^b^	−13.1 ± 13.8^c^	−23.6 ± 10.8	−26.3 ± 11.4
FN-BDR (%)	−3.07 ± 12.5^b^	−10.2 ± 14.5^c^	−21.1 ± 11.1^d^	−27.4 ± 9.85
Hip-BDR (%)	−2.34 ± 12.2^b^	−9.96 ± 13.7^c^	−18.6 ± 10.8^d^	−25.0 ± 10.1
RUUD-BDR (%)	−1.02 ± 9.62^b^	−9.61 ± 13.6^c^	−24.1 ± 10.9^d ^	−34.1 ± 10.3^e^

OPG: osteoprotegerin, TGF-*β*: transforming growth factor-beta, BDR: bone mineral density decrease rate, PA: posteroanterior spine, FN: femoral neck, Hip: total hip, and RUUD: radius + ulna ultradistal.

^a^Values are geometric mean ± SD.

^b^ *P* = 0.012–0.000 compared with 45–54, 55–64 and ≥65-year age groups.

^c^ *P* = 0.049–0.000 compared with 55–64 and ≥65-year age groups.

^d^ *P* = 0.011–0.000 compared with ≥65-year age group.

^e^ *P* = 0.003–0.000 compared with other sites in the same age group.

**Table 3:** Correlation coefficients (*r*) for serums OPG, TGF-*β*1, and TGF-*β*2 with BDR at various skeletal sites in native Chinese women.

Marker	OPG		TGF-*β*1		TGF-*β*2	
	*r*	P-*r *	*r*	P-*r *	*r*	P-*r *
TGF-*β*1	−0.270^a^	−0.215^a^				
TGF-*β*2	0.433^a^	0.399^a^	−0.237^a^	−0.172^a^		
PA-BDR	−0.230^a^	−0.044	0.365^a^	0.183^a^	−0.173^a^	−0.030
FN-BDR	−0.122^a^	0.066	0.245^a^	0.014	−0.110^a^	0.026
Hip-BDR	−0.148^a^	0.040	0.276^a^	0.080	−0.100^a^	0.040
RUUD-BDR	−0.205^a^	−0.024	0.344^a^	0.104^a^	−0.145^a^	0.020

Pearson's correlation coefficients (*r*) and partial correlation coefficients (P-*r*) after adjustment for age and body mass index are shown.

OPG: osteoprotegerin, TGF-*β*: transforming growth factor-beta, BDR: bone mineral density decrease rate, PA: posteroanterior spine, FN: femoral neck, Hip: total hip, and RUUD: radius + ulna ultradistal.

^a^ *P* = 0.036–0.000.

**Table 4:** Multiple linear regression analysis of OPG, TGF-*β*1, and TGF-*β*2 with BDR at various skeletal sites in native Chinese women.

Dependent	OPG		TGF-*β*1		TGF-*β*2	
	**β**	*R* ^2^C (%)	**β**	*R* ^2^C (%)	**β**	*R* ^2^C (%)
PA-BDR	−0.179^a^	2.9	0.309^a^	13.3	—^b^	—^b^
FN-BDR	−0.127^a^	1.4	0.190^a^	5.3	—^b^	—^b^
Hip-BDR	−0.138^a^	1.7	0.229^a^	7.4	—^b^	—^b^
RUUD-BDR	−0.202^a^	3.7	0.266^a^	10.8	—^b^	—^b^

Serums OPG, TGF-*β*1, and TGF-*β*2 were independent variables; BDRs were dependent variables.

OPG: osteoprotegerin, TGF-*β*: transforming growth factor-beta, BDR: bone mineral density decrease rate, *R* ^2^C: *R* square change, PA: posteroanterior spine, FN: femoral neck, Hip: total hip, and RUUD: radius + ulna ultradistal.

^a^ *P* = 0.012–0.000.

^b^Independent was excluded in this analysis.
